# The peritoneal immune niche in peritoneal dialysis-induced membrane failure: macrophages, neutrophils and extracellular trap biology

**DOI:** 10.3389/fimmu.2026.1875068

**Published:** 2026-08-20

**Authors:** Yuqing Lu, Jasmin Knopf, Martin Herrmann, Leyi Gu, Michael Boettcher, Julia Elrod

**Affiliations:** 1 Department of Pediatric Surgery, University Medical Center Mannheim, Medical Faculty Mannheim, Heidelberg University, Mannheim, Germany; 2 European Center for Angioscience (ECAS), Medical Faculty Mannheim, Heidelberg University, Mannheim, Germany; 3 Mannheim Institute for Innate Immunoscience (MI3), Medical Faculty Mannheim, Heidelberg University, Mannheim, Germany; 4 Department of Rheumatology and Immunology, West China Hospital, Sichuan University, Chengdu, China; 5 Department of Nephrology, Molecular Cell Lab for Kidney Disease, Shanghai Peritoneal Dialysis Research Center, Renji Hospital, Shanghai Jiao Tong University, School of Medicine, Shanghai, China

**Keywords:** cGAS–STING, encapsulating peritoneal sclerosis, macrophage extracellular traps, macrophages, neutrophil extracellular traps, peritoneal dialysis, peritoneal fibrosis, ultrafiltration failure

## Abstract

Peritoneal dialysis (PD) is associated with progressive remodeling of the peritoneal membrane, with fibrosis, vasculopathy, and transport deterioration that culminate in ultrafiltration failure (UFF); a smaller subset of patients progresses to encapsulating peritoneal sclerosis (EPS). Myeloid-driven sterile inflammation is a central feature of this process, yet the evidence base differs sharply across the immune actors implicated. In this Perspective, we evaluate that evidence through a three-layer framework separating PD-direct data from non-PD peritoneal injury data and other-organ analogies. Macrophages are the best-established organizers of peritoneal niche remodeling. Neutrophils are well characterized in peritonitis but only gradually appear in chronic non-infectious functional disorders. Among extracellular traps, neutrophil extracellular traps (NETs) carry the strongest PD-specific signal: effluent proxies are linked to membrane transport phenotype and risk for technical failure. Macrophage extracellular traps (METs) rest on a single PD-specific mechanistic study and preliminary conference-level human data. We propose that chronic PD exposure conditions this niche and that second-hit inflammatory events (such as peritonitis) accelerate injury. If extracellular trap formation proceeds faster than nuclease- and macrophage-mediated clearance, trap-derived extracellular DNA may persist in the peritoneal cavity. We further hypothesize that, after uptake by bystander cells, cytosolic DNA sensing through cGAS–STING is engaged and may amplify membrane failure.

## Graphical Abstract

**Figure:**
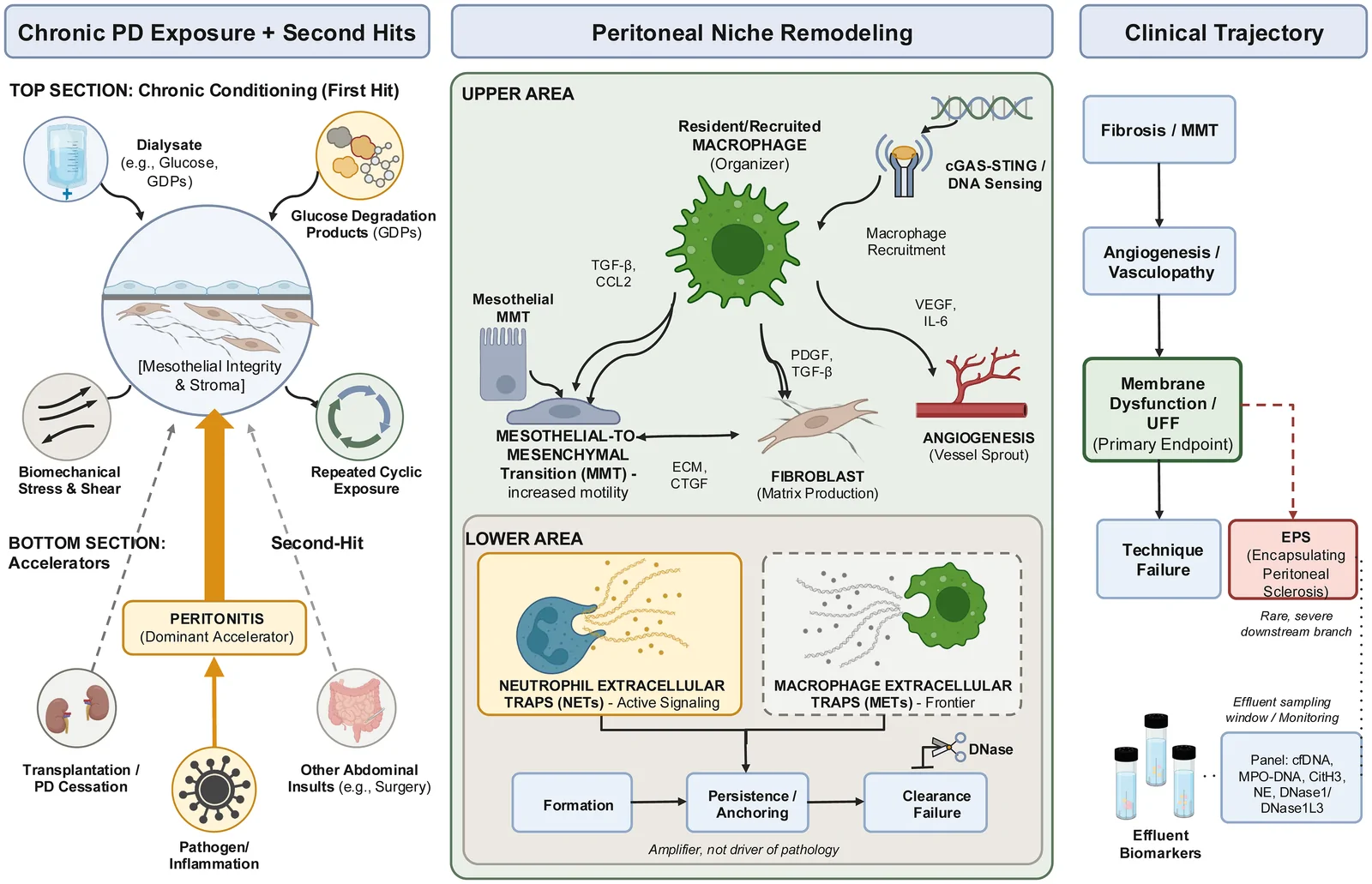

## 1 Introduction

Peritoneal dialysis (PD) is a home-based renal replacement therapy that uses the peritoneum as a semipermeable membrane for solute exchange and ultrafiltration. With time, repeated exposure to glucose-based, bioincompatible dialysis solutions remodel the peritoneal membrane. Mesothelial injury, mesothelial-to-mesenchymal transition (MMT), submesothelial fibrosis, angiogenesis, and vasculopathy progressively alter transport behavior and cause ultrafiltration failure (UFF) (1, 2). UFF affects 30–50% of patients after 6 years of PD and is responsible for approximately 25% of non-infection-related technique failure. A smaller subset develops encapsulating peritoneal sclerosis (EPS), a rare (0.7–7.3% incidence) but serious complication with a one-year survival rate of less than 50% in some cohorts (3, 4).

Long-term PD exposure acts less as a one-time insult than as chronic conditioning of the peritoneal immune–mesothelial–stromal niche. On this preconditioned background, second-hit events, often peritonitis, accelerate progression toward severe downstream phenotypes including EPS. PD membrane failure has long been attributed to TGF-β/Smad signaling (5); but it may be better understood as a systems-level disorder of the peritoneal immune niche, in which myeloid cells and their extracellular DNA-containing effector structures have emerged as candidate organizers of disease rather than secondary participants.

Extracellular traps are web-like chromatin scaffolds first described in neutrophils (6) and later in macrophages and other myeloid lineages (7). They can amplify sterile inflammation, injure bystander cells through histones and proteases, and engage cytosolic DNA sensors (8, 9). Outside the peritoneal compartment, extracellular traps are implicated in fibrotic remodeling in the lung (10), liver (11), and kidney (12). Whether this cross-organ principle operates under chronic PD exposure is discussed here.

Within PD itself, macrophages are the best-established actors and neutrophils are transitioning from peritonitis biology into UFF-associated microenvironments. Evidence for extracellular traps is scarce and unevenly distributed. While PD-specific NETs reports are strong, the PD-specific MET findings remain limited (**Table 1**). Our central thesis is that chronic PD exposure conditions the peritoneal niche, superimposes inflammatory insults, and accelerates injury. When extracellular trap formation proceeds faster than clearance, extracellular DNA that is internalized by bystander cells may engage cytosolic cGAS–STING signaling and, we propose, amplify downstream membrane failure.

**Table 1:** Evidence overview: four myeloid actors across PD-relevant endpoints.

Actor	PD-direct	PD-indirect	Other-organ analogy	Fibrosis/MMT	Angiogenesis/vasculopathy	UFF/membrane failure	Technique failure	EPS
Macrophages	Established (14, 15, 17, 22)	No PD data	Emerging (11)	Established (14, 17)	Emerging (14)	Emerging (22)	No PD data	Preliminary (16)
Neutrophils	Established for acute peritonitis (19, 20)	Emerging (26)	Emerging (10, 12)	Analogy only (10, 12)	Emerging (23)	No PD data	Emerging (mostly via NETs) (21)	No PD data
NETs	Established, association (adult cohort + pediatric tissue) (21, 23)	Established, includes DNase intervention (26, 27)	Emerging (10, 12, 13)	Emerging (23)	Emerging (23)	Emerging (21)	Emerging (21)	No PD data
METs	Preliminary (28–30)	No PD data	Emerging (11)	Preliminary (28)	Preliminary (28)	No PD data	No PD data	Preliminary, conference data only (29, 30)

Evidence levels follow the criteria below and refer to support within the stated tier. Tier definitions follow Section 2.

Established — two or more independent human cohorts agreeing in direction, or one human cohort plus interventional evidence in a PD or peritoneal model.

Emerging — one human cohort of any design, or two or more concordant animal or in vitro studies in a PD or peritoneal system.

Preliminary — a single animal or in vitro PD study, or human data available only in abstract form.

Analogy only — no peritoneal data; inference drawn from another organ.

No PD data — no evidence retrieved in this tier.

Levels reflect strength of published association, not analytical certainty. MPO–DNA ELISA has documented performance limitations in plasma (24). Levels reflect literature available as of July 2026.

## 2 A three-layer evidence framework

To avoid conflating mechanistic plausibility with PD-specific demonstration, we apply a three-layer evidence framework throughout. PD-direct evidence comes from cohort studies with human PD, PD patient biopsies, and experimental models of PD fluid exposure. PD-indirect evidence comes from non-PD peritoneal injury, particularly post-surgical peritoneal adhesions. These share mesothelial and stromal biological similarities with PD, but without coming into contact with dialysate fluid. Other-organ analogy evidence includes extracellular-trap and myeloid-fibrosis biology in lung, liver, kidney, or vasculature and supports plausibility but does not constitute a PD-specific proof. Grading criteria within each layer are stated in the legend to **Table 1**. Other-organ analogy evidence contributes to plausibility but cannot upgrade PD-specific grades. These three tiers are applied consistently in **Table 1**. This is not a systematic review; it is a critical synthesis informed by the authors’ expertise in pediatric peritoneal surgery, innate immunity, and adult peritoneal dialysis, with conference papers and preprints identified as such. While mesothelial defense mechanisms, T-lymphocyte responses, and soluble inflammatory mediators also shape the peritoneal immune niche, we focus on the myeloid-extracellular trap axis where current PD-specific evidence is most concentrated, and intervention targets are most feasible.

## 3 The peritoneal niche and the macrophage axis

The peritoneum is an immunologically active serosal organ in which mesothelial cells serve as both a physical barrier and a signaling hub. Chronic PD stress includes hyperosmolar glucose, glucose degradation products (GDPs), biomechanical distension, low-pH lactate buffering, and recurrent inflammation. Under these conditions, mesothelial chemokine production, damage-associated molecular pattern (DAMP) release, and progressive barrier disruption favor immune recruitment, fibroblast activation, and matrix remodeling (5). Extracellular traps may act as amplifiers within this niche. Extracellular DNA, histones, and proteases reinforce DAMP signaling and after engulfment engage cytosolic cGAS–STING sensing (8, 9, 13).

Macrophages serve as the best-established immune organizers of this remodeling. Repeated PD-fluid exposure progressively erodes resident peritoneal macrophage populations and promotes their replacement by profibrotic monocyte-derived macrophage (14, 15). GDP-containing fluids intensify this shift, with near-complete depletion of resident peritoneal macrophages in experimental models as well as stronger TGF-β and interferon-associated signaling. These findings indicate homeostatic erosion *plus* pathological reprogramming, rather than mere exhaustion. Macrophages likely occupy a central node where dialysate-driven mesothelial injury, neutrophil-derived DAMPs, and fibroblast activation converge, rather than orchestrating fibrosis alone.

The strongest PD/EPS-specific macrophage recruitment mechanism sits in the STING-CCL2 branch. In a PD-induced EPS model, mesothelial STING activation increases CCL2 production, macrophage infiltration, collagen deposition, and adhesion severity; the STING inhibitor H151 attenuated each readout (16). An independent study extended this to macrophage STING as blocking STING in activated macrophages prevented MMT induction in co-cultured mesothelial cells, and STING inhibition *in vivo* reduced peritoneal inflammation, peritonitis severity, and post-surgical adhesion (17). DNA sensing therefore appears to operate downstream of peritoneal injury and upstream of macrophage-mediated fibrotic progression. Here it mechanistically bridges extracellular-trap biology and macrophage-centric remodeling that we develop in Section 7. Clinical and pediatric data further indicate that PD duration and glucose exposure are associated with MMT (18).

## 4 Neutrophils: from peritonitis toward chronic remodeling

Neutrophils are well characterized in acute peritonitis but their role in chronic, non-infectious membrane dysfunction is less clear. Currently, there is increasing evidence of chronic remodeling processes in UFF-associated microenvironments (13, 19). PD peritonitis is a neutrophil-dominated response in which mesothelial IL-6 production and sIL-6R-mediated trans-signaling eventually orchestrates the switch from early neutrophil influx to mononuclear recruitment (20). This is one of the best-characterized resolution programs in serosal biology, and a benchmark against which chronically disordered PD responses can be compared.

In a prospective adult PD cohort that quantified NET-associated readouts, acute peritonitis samples showed markedly higher nucleosome and myeloperoxidase (MPO)–DNA levels than noninfectious PD effluent (21). Even without overt infection, these signals correlated with monocyte chemoattractant protein (MCP)-1 and matrix metalloproteinase-8, suggesting that neutrophil-linked extracellular programs persist in noninfectious states of ongoing peritoneal injury. In a single-cell study of 16 long-term PD patients, effluent neutrophils were numerically but not significantly higher with ultrafiltration failure, while MRC1-macrophages declined and monocyte/macrophage transcriptomes shifted toward inflammatory programs (22). Pediatric longitudinal profiling adds tissue-level support, with prominent NET-like chromatin structures accompanying chronic PD exposure (detailed in Section 5) (23).

Neutrophils therefore occupy an intermediate position: established in acute peritonitis, emerging in chronic remodeling, but not yet as causal drivers of PD fibrosis or MMT in the same way as macrophages are. What is more strongly related to the membrane phenotype than the number of neutrophils alone are the extracellular DNA protease complexes they release. This distinction matters for intervention (Section 8.3): targeting extracellular effector structures leaves innate antimicrobial function largely intact, whereas neutrophil depletion would not.

## 5 NETs: the strongest PD-specific extracellular traps

NETs provide the most established PD-specific extracellular-trap readout, according to two complementary PD-direct sources. In a prospective cohort of 250 patients, higher effluent NET proxies were associated with a higher 4-hour dialysate-to-plasma (D/P) creatinine, a smaller 1-hour sodium dip, and an approximately 1.9-fold risk of technique failure (95% CI 1.27–2.83) (21). Acute peritonitis samples showed the highest nucleosome and MPO–DNA levels; notably, NET-associated signal was also detectable in the absence of overt infection. In the three-pore theory of peritoneal transport (1, 2), higher 4-h D/P creatinine reflects faster small-solute transport and reduced 1-h sodium dip indicates impaired aquaporin-1-mediated free-water transport. NET-associated proxies capture these functional phenotypes in associational analyses, but no PD study has yet demonstrated that altering extracellular trap formation or clearance restores three-pore parameters. Membrane failure here was captured by transport phenotype, not by measured ultrafiltration volume.

The pediatric study moves from effluent association toward tissue remodeling. After chronic PD exposure, pediatric peritoneal samples showed prominent NET-like chromatin structures, marked immune-cell infiltration, doubled microvessel density, tripled submesothelial thickness, and two- to fourfold increases in dialysate and plasma NET-associated components (23). DNase1 and DNase1L3 expression levels further suggest that extracellular DNA persistence and incomplete clearance accompany structural remodeling. Consistent with this, chromatin scaffolds can concentrate VEGF and matrix metalloproteinases in vascular beds, positioning NETs as plausible amplifiers at the inflammation–angiogenesis interface. Pediatric-to-adult extrapolation requires caution: the pediatric peritoneum is immunologically more permissive and less confounded by diabetes-related microvascular disease, which may strengthen the dataset as a mechanistic reference point while limiting direct magnitude translation.

NET biology in PD is best described as imbalance between formation, persistence, and clearance. The adult cohort emphasizes persistent NET-associated signal in noninfectious effluent; the pediatric data point toward incomplete extracellular DNA clearance during remodeling. Clearance failure may be dual: enzymatic (reduced DNase1/DNase1L3 activity) and cellular (impaired macrophage efferocytosis). As chronic PD erodes macrophage homeostatic function (Section 3), both arms may be compromised simultaneously. Whether routine dialysate exchange clears surface-anchored chromatin is untested; soluble fragments are likely flushed, but chromatin anchored to injured mesothelial surfaces through histone–matrix interactions is probably not. If validated, this mechanism would integrate NET biology directly into the three-pore framework.

Methodological caution remains essential. PD effluent is a low-biomass, high-variance matrix. Current readouts like MPO–DNA complexes, citrullinated histone H3 (CitH3), nucleosome, or cfDNA should therefore be interpreted as NET-associated proxies rather than standardized NET biomarkers (24, 25). Cell-free DNA is not NET-specific. MPO–DNA complexes are more specific but still correlative, and on their own cannot show that NETs drive the pathology. Post-surgical peritoneal adhesion biology, while compartment-matched and instructive (26, 27), cannot establish PD facts by analogy: PD is chronic, cyclic, and transport-defined, whereas adhesions follow a discrete injury-and-repair course.

## 6 METs: limited PD-specific evidence

The PD-direct MET evidence base remains thin. It is based on a single 2025 mechanistic study in a high-glucose dialysate model (28), with additional human findings on EPS limited to two conference abstracts (29, 30). Under high-glucose conditions, MET formation was linked to reactive oxygen species (ROS), TGF-β, and Smad signaling (28). This describes a PD-relevant mechanism involving METs in a single model, rather than one derived solely from other organs. At present there is no independent replication, no human peritoneal tissue confirmation, and no validated MET biomarker in PD.

Two technical obstacles constrain progress in the field. First, MET identification remains methodologically difficult: macrophages do not decondense chromatin with the reproducibility of neutrophils, and co-staining strategies (CD68/CD163 with CitH3 and extracellular DNA) are sensitive to fixation and imaging. Second, macrophage heterogeneity means that MET-prone states must be defined in context, not as a fixed phenotype. METs therefore remain a preliminary component of this framework rather than a validated biomarker or therapeutic target. Until independent replication and human peritoneal tissue confirmation are available, MET involvement in PD membrane failure should be regarded as a working hypothesis rather than an established mechanism.

## 7 NET–MET crosstalk and the cGAS–STING amplifier: our hypothesis

We hypothesize that a fragmented view of neutrophil- and macrophage-derived traps underestimates their coupling in the PD peritoneum. In non-PD settings, NET-derived DAMPs (histones, HMGB1, oxidized mitochondrial DNA) license macrophage cytokine production, prime inflammasome activity, and shift macrophages toward states compatible with release of extracellular DNA traps (9). In the peritoneal context, we outline a plausible but presently unproven sequence. First, early mesothelial injury and peritonitis recruit neutrophils that deposit NETs (PD-direct, emerging). Second, incomplete DNase- and efferocytosis-mediated clearance leaves extracellular chromatin on the serosal surface (emerging). Third, recruited and reprogrammed macrophages encounter this persistent ligand load, and cytosolic DNA sensing may lower the threshold for MET release (speculative in PD). This positions NET persistence upstream of MET generation rather than as a parallel process.

The cGAS–STING axis provides the best-defined molecular bridge currently available in PD. cGAS is a cytosolic double-stranded DNA sensor. Extracellular chromatin fragments must be internalized by bystander cells before engaging this pathway, and the resulting STING signaling induces type I interferon and chemokine production, as shown for NETs in non-PD systems (9). Several upstream nodes of this circuit have been described in PD. Chronic PD exposure erodes resident peritoneal macrophage homeostasis, including efferocytic capacity (15). PD effluent contains measurable cell-free DNA, with levels varying by dialysate biocompatibility and rising during peritonitis (31, 32). HMGB1 follows a similar pattern in peritonitis effluent, tracking inflammatory cytokine load (33). Two independent studies now show that pharmacological STING blockade reduces macrophage infiltration, MMT, and adhesion in PD-relevant models, implicating both mesothelial and macrophage STING (16, 17). What no PD study has yet defined is the upstream DNA source that drives this activation.

We hypothesize that NET- and MET-derived chromatin is a major source. Two additional inputs may amplify this load: nuclear DNA when mesothelial and immune cells undergo secondary necrosis after failed efferocytosis, and DNA from necroptotic stromal cells. Once internalized, this combined load may sustain a cGAS–STING–CCL2 loop that could reinforce macrophage infiltration and fibrosis. The link from efferocytosis failure to cGAS–STING activation has not been tested in PD by genetic or pharmacological intervention and is a high-priority test of the model. Whether the non-canonical cGAS–STING–PERK branch, which couples DNA sensing to senescence and organ fibrosis (34), also contributes through senescence‐associated secretory phenotype (SASP)-mediated paracrine signaling remains similarly open.

Extracellular DNA capable of engaging cGAS–STING is not restricted to extracellular traps. Lytic forms of regulated cell death mobilize DNA that reaches this pathway, as shown for MLKL-dependent necroptosis (35). The peritoneal cavity is no exception. Cell-free DNA and mitochondrial DNA are measurable in effluent and track with intraperitoneal inflammation and solute transport (31, 32, 36), glucose-based dialysate damages mesothelial mitochondrial DNA (37), and STING signaling is upregulated in experimental peritoneal damage and in patient biopsies without the source DNA being identified (17). Trap-derived and non-trap DNA are also not cleanly separable, since NETs themselves can be enriched in oxidized mitochondrial DNA (38). We therefore do not position traps as the dominant source of peritoneal extracellular DNA. Among the candidates, they are the one that can currently be quantified in effluent and targeted with available agents, which is what makes this arm of the model testable first. During peritonitis, pathogen-derived DNA adds a further contribution (39).

Three falsifiable predictions follow from this model, each directly testable with current PD resources. If this model holds:

a. In pediatric and adult effluent cohorts, markers of impaired efferocytosis (MerTK shedding, soluble TIM-4) should co-vary longitudinally with extracellular chromatin load (cfDNA, MPO–DNA, HMGB1) and with downstream CCL2. Peritonitis episodes amplify these correlations.
b. Tissue co-localization of citrullinated histone H3 with CD68/CD163 and phosphorylated STING should be more frequent in UFF and EPS biopsies than in early PD exposure.
c. Pharmacological STING inhibition in trap-rich PD models should attenuate downstream fibrotic endpoints disproportionately to peritonitis incidence, distinguishing DNA-sensing amplification from antimicrobial trap function.

## 8 Translational implications

### 8.1 PD effluent as a uniquely accessible biomarker compartment

PD allows repeated access to the disease compartment itself: serial effluent sampling can be aligned with transport tests, peritonitis episodes, and technique outcomes. NET-associated proxies such as cfDNA, CitH3/NE/MPO, and DNase1/DNase1L3 are well-suited as longitudinal compartment-level markers. They meet the threshold for biomarker plausibility but not for full clinical-laboratory validation (24). No replicated human PD effluent MET platform exists. For effluent-based biomarkers to move from mechanistic probes to clinical tools, the field needs multi-center assay harmonization, pre-analytical standards, and prospective linkage to hard PD endpoints rather than surrogate transport parameters alone.

### 8.2 Intervention hierarchy: DNase, PAD, and DNA-sensing

The intervention landscape has three entry points: chromatin dismantling/clearance (DNase), upstream control of trap formation (Protein-arginine deiminase (PAD), ROS), and downstream DNA-sensing blockade (STING). Their bases of evidence differ. PAD- and ROS-linked strategies act earlier but PD-specific evidence is limited. DNase as dornase alfa is already in clinical use, even though not for PD. Among downstream targets, STING blockade has the strongest PD-direct support. Two independent studies show that STING inhibition reduces peritoneal fibrosis, MMT, and adhesion in PD-relevant models (16, 17).

### 8.3 Anti-infective safety as a design constraint

PD peritonitis remains the dominant cause of technique failure and a meaningful contributor to PD-related mortality. Any therapy that reduces peritoneal neutrophil function, trap formation, or chromatin clearance must be evaluated against its potential to increase peritonitis incidence, severity, or pathogen spectrum. This constraint shapes the intervention hierarchy: downstream DNA-sensing blockade (STING) has a more favorable theoretical safety profile than upstream NET/MET formation blockade, because it would be expected to preserve trap-dependent antimicrobial function while dampening fibrotic amplification. This remains a theoretical advantage; no comparative safety data exist in PD. DNase-based clearance enhancement occupies an intermediate position. Any clinical trial of trap-modulating therapy in PD should pre-specify peritonitis incidence as a co-primary safety endpoint, independent of the efficacy endpoint under investigation.

### 8.4 Where trap biology most likely matters

On current evidence, the patient groups most likely to show measurable benefit are long-term PD patients with high-transporter status and reduced 1-h sodium dip; pediatric patients with rapid submesothelial thickening and microvascular expansion; and patients with clinically or radiologically suspected early EPS. Patients with recent or recurrent peritonitis require particular caution and should be analyzed separately. Prospective trial design should incorporate these groups from the outset rather than as *post-hoc* subgroups.

## 9 Outlook

PD membrane failure reflects chronic peritoneal niche remodeling rather than fibrosis alone. Macrophages remain the established organizing axis, neutrophils define the acute-to-chronic interface, NETs offer the most clinically anchored trap signal, and the PD-specific METs remain the most limited. Chronic PD exposure conditions the niche, later inflammatory events accelerate injury, and NET/MET biology, coupled through a cGAS–STING amplifier, is one possible mechanistic interface within this trajectory. The three predictions in Section 7 make this Perspective falsifiable, and the patient strata in Section 8.4 make it actionable. The goal is not a trap-only theory of PD but a more precise account of how chronic sterile inflammation contributes to durable membrane failure.

## Data Availability

The original contributions presented in the study are included in the article/supplementary material. Further inquiries can be directed to the corresponding author.

## References

[1] Morelle J Stachowska-Pietka J Öberg C Gadola L La Milia V Yu Z . Ispd recommendations for the evaluation of peritoneal membrane dysfunction in adults: classification, measurement, interpretation and rationale for intervention. Perit Dial Int. (2021) 41(4):352–72. doi: 10.1177/0896860820982218 33563110

[2] Krediet RT . Acquired decline in ultrafiltration in peritoneal dialysis: the role of glucose. J Am Soc Nephrol. (2021) 32:2408–15. doi: 10.1681/asn.2021010080 34321252 PMC8722789

[3] Kawanishi H Moriishi M . Epidemiology of encapsulating peritoneal sclerosis in Japan. Perit Dial Int. (2005) 25:S14–8. doi: 10.1177/089686080502504s03 16300268

[4] Petrie MC Traynor JP Mactier RA . Incidence and outcome of encapsulating peritoneal sclerosis. Clin Kidney J. (2016) 9:624–9. doi: 10.1093/ckj/sfw051 27478609 PMC4957727

[5] Yanez-Mo M Lara-Pezzi E Selgas R . Peritoneal dialysis and epithelial-to-mesenchymal transition of mesothelial cells. N Engl J Med. (2003) 348:403–13. doi: 10.1056/NEJMoa020809 12556543

[6] Brinkmann V Reichard U Goosmann C Fauler B Uhlemann Y Weiss DS . Neutrophil extracellular traps kill bacteria. Science. (2004) 303:1532–5. doi: 10.1126/science.1092385 15001782

[7] Papayannopoulos V . Neutrophil extracellular traps in immunity and disease. Nat Rev Immunol. (2018) 18:134–47. doi: 10.1038/nri.2017.105 28990587

[8] Xu J Zhang X Pelayo R Monestier M Ammollo CT Semeraro F . Extracellular histones are major mediators of death in sepsis. Nat Med. (2009) 15:1318–21. doi: 10.1038/nm.2053 19855397 PMC2783754

[9] Apel F Andreeva L Knackstedt LS Streeck R Frese CK Goosmann C . The cytosolic DNA sensor cGAS recognizes neutrophil extracellular traps. Sci Signaling. (2021) 14:eaax7942. doi: 10.1126/scisignal.aax7942 33688080

[10] Chrysanthopoulou A Mitroulis I Apostolidou E . Neutrophil extracellular traps promote differentiation and function of fibroblasts. J Pathol. (2014) 233:294–307. doi: 10.1002/path.4359 24740698

[11] Cao CJ Fang X Wang MM . Hepatocyte-derived IL-25 promotes macrophage extracellular trap formation and drives liver fibrosis progression. Inflammation. (2026) 49:139. doi: 10.2139/ssrn.5583381 41922835 PMC13171947

[12] Wang Y Li Y Chen Z . GSDMD-dependent neutrophil extracellular traps promote macrophage-to-myofibroblast transition and renal fibrosis in obstructive nephropathy. Cell Death Dis. (2022) 13:693. doi: 10.1038/s41419-022-05138-4 35941120 PMC9360039

[13] Warnatsch A Ioannou M Wang Q Papayannopoulos V . Neutrophil extracellular traps license macrophages for cytokine production in atherosclerosis. Science. (2015) 349:316–20. doi: 10.1126/science.aaa8064 26185250 PMC4854322

[14] Bellon T Martinez V Lucendo B del Peso G Castro MJ Aroeira LS . Alternative activation of macrophages in human peritoneum: implications for peritoneal fibrosis. Nephrol Dialysis Transplant. (2011) 26:2995–3005. doi: 10.1093/ndt/gfq771 21324976

[15] Sutherland TE Shaw TN Lennon R Herrick SE Ruckerl D . Ongoing exposure to peritoneal dialysis fluid alters resident peritoneal macrophage phenotype and activation propensity. Front Immunol. (2021) 12:715209. doi: 10.3389/fimmu.2021.715209 34386014 PMC8353194

[16] Sun J Sun Y Guo D . Targeting STING to disrupt macrophage-mediated adhesion in encapsulating peritoneal sclerosis. Commun Biol. (2025) 8:1266. doi: 10.1038/s42003-025-08662-z 40847090 PMC12373792

[17] Marchant V García-Giménez J González-Mateo GT Sandoval P Tejedor-Santamaria L Rayego-Mateos S . STING inhibition alleviates experimental peritoneal damage: potential therapeutic relevance for peritoneal dialysis. J Pathol. (2025) 267:196–212. doi: 10.1002/path.6462 40810380 PMC12438029

[18] Bartosova M Schaefer B Vondrak K . Peritoneal dialysis vintage and glucose exposure but not peritonitis episodes drive peritoneal membrane transformation during the first years of PD. Front Physiol. (2019) 10:356. doi: 10.3389/fphys.2019.00356 31001140 PMC6455046

[19] McLoughlin RM . Resolving peritoneal inflammation: flicking the right 'switches'. Peritoneal Dialysis Int. (2005) 25:223–9. doi: 10.1177/089686080502500303 15981768

[20] Hurst SM Wilkinson TS McLoughlin RM Jones S Horiuchi S Yamamoto N . IL-6 and its soluble receptor orchestrate a temporal switch in the pattern of leukocyte recruitment seen during acute inflammation. Immunity. (2001) 14:705–14. doi: 10.1016/s1074-7613(01)00151-0 11420041

[21] Kim I Min SH Lee HW . Impact of peritoneal neutrophil extracellular traps on peritoneal characteristics and technical failure in patients undergoing peritoneal dialysis. Am J Nephrol. (2025) 56:136–47. doi: 10.1159/000542427 39510040

[22] Diao X Zhan C Ye H . Single-cell transcriptomic reveals the peritoneal microenvironmental change in long-term peritoneal dialysis patients with ultrafiltration failure. iScience. (2024) 27:111383. doi: 10.1016/j.isci.2024.111383 39687014 PMC11647153

[23] Dücker CM Herrmann M Boettcher S Bauer-Carmona S Schild RS Schönfeld L . Neutrophil extracellular traps drive peritoneal inflammation and tissue remodeling in pediatric peritoneal dialysis. Pediatr Nephrol. (2026) 41(3):819–29. doi: 10.1007/s00467-025-07003-w PMC12852172 41108377

[24] Hayden H Ibrahim N Klopf J . ELISA detection of MPO-DNA complexes in human plasma is error-prone and yields limited information on neutrophil extracellular traps formed *in vivo*. PloS One. (2021) 16:e0250265. doi: 10.1371/journal.pone.0250265 33886636 PMC8062102

[25] Morisi N . Exploring the role of cell-free nucleic acids and peritoneal dialysis: a narrative review. Genes. (2024) 15:553. doi: 10.3390/genes15050553 38790182 PMC11121405

[26] Elrod J Heuer A Knopf J Schoen J Schönfeld L Trochimiuk M . Neutrophil extracellular traps and DNases orchestrate formation of peritoneal adhesions. iScience. (2023) 26(12):108289. doi: 10.2139/ssrn.4336831 38034352 PMC10682263

[27] Lu Y Elrod J Herrmann M Knopf J Boettcher M . Neutrophil extracellular traps: a crucial factor in post-surgical abdominal adhesion formation. Cells. (2024) 13:991. doi: 10.3390/cells13110991 38891123 PMC11171752

[28] Wang J Yu C Lyu H Liu T Xie R . Macrophage extracellular traps promote peritoneal fibrosis through the ROS/TGF-beta/Smad pathway under high-glucose dialysis conditions. Int Immunopharmacol. (2025) 167:115748. doi: 10.1016/j.intimp.2025.115748 41151485

[29] Sun J Peng H . Mechanism of macrophage extracellular traps involved in the formation of encapsulating peritoneal sclerosis: SA-PO453. J Am Soc Nephrol. (2024) 35:10.1681/ASN.2024xzb5bqdk. doi: 10.1681/asn.2024xzb5bqdk

[30] Sun Y Peng H . WCN26-1281 Single cell and spatial transcriptomics reveal macrophage extracellular trap as pivotal driver of encapsulated peritoneal sclerosis progression. Kidney Int Rep. (2026) 11:105839. doi: 10.1016/j.ekir.2026.105839 38826717

[31] Pajek J Kveder R Gucek A Skoberne A Bren A Bucar M . Cell-free DNA in the peritoneal effluent of peritoneal dialysis solutions. Ther Apher Dial. (2010) 14:20–6. doi: 10.1093/ndtplus/4.s2.54 20438516

[32] Virzì GM Milan Manani S Brocca A Cantaluppi V de Cal M Pastori S . Peritoneal Cell-free DNA: an innovative method for determining acute cell damage in peritoneal membrane and for monitoring the recovery process after peritonitis. J Nephrol. (2016) 29(1):111–8. doi: 10.1007/s40620-015-0212-2 26012380

[33] Cao S Li S Li H Xiong L Zhou Y Fan J . The potential role of HMGB1 release in peritoneal dialysis-related peritonitis. PloS One. (2013) 8:e54647. doi: 10.1371/journal.pone.0054647 23359306 PMC3554653

[34] Zhang D Liu Y Zhu Y Zhang Q Guan H Liu S . A non-canonical cGAS-STING-PERK pathway facilitates the translational program critical for senescence and organ fibrosis. Nat Cell Biol. (2022) 24:766–82. doi: 10.1038/s41556-022-00894-z 35501370

[35] Ding Z . MLKL activates the cGAS-STING pathway by releasing mitochondrial DNA upon necroptosis induction. Mol Cell. (2025) 85:2610–25. doi: 10.1016/j.molcel.2025.06.005 40614706

[36] Xie X Wang J Xiang S Chen Z Zhang X Chen J . Dialysate cell-free mitochondrial DNA fragments as a marker of intraperitoneal inflammation and peritoneal solute transport rate in peritoneal dialysis. BMC Nephrol. (2019) 20:128. doi: 10.1186/s12882-019-1284-3 30975091 PMC6458606

[37] Ishibashi Y Sugimoto T Ichikawa Y Akatsuka A Miyata T Nangaku M . Glucose dialysate induces mitochondrial DNA damage in peritoneal mesothelial cells. Peritoneal Dialysis Int. (2002) 22:11–21. doi: 10.1177/089686080202200103 11929138

[38] Lood C Blanco LP Purmalek MM Carmona-Rivera C De Ravin SS Smith CK . Neutrophil extracellular traps enriched in oxidized mitochondrial DNA are interferogenic and contribute to lupus-like disease. Nat Med. (2016) 22:146–53. doi: 10.1038/nm.4027 26779811 PMC4742415

[39] Szeto C-C Lai K-B Kwan B-H Chow K-M Leung C-B Law M-C . Bacteria-derived DNA fragment in peritoneal dialysis effluent as a predictor of relapsing peritonitis. Clin J Am Soc Nephrol. (2013) 8:1935–41. doi: 10.2215/cjn.02360213 24092821 PMC3817904

